# Persistent high fertility in Uganda: young people recount obstacles and enabling factors to use of contraceptives

**DOI:** 10.1186/1471-2458-10-530

**Published:** 2010-09-03

**Authors:** Gorrette Nalwadda, Florence Mirembe, Josaphat Byamugisha, Elisabeth Faxelid

**Affiliations:** 1 Department of Nursing, College of Health Sciences Makerere University P.O Box 7072, Kampala, Uganda; 2 Department of Obstetrics and Gynecology, College of Health Sciences Makerere University, P.O Box 7072, Kampala Uganda; 3 Division of Global Health (IHCAR), Department of Public Health Sciences, Karolinska Institutet, Nobels vag 9 S-171, Stockholm, Sweden

## Abstract

**Background:**

High fertility among young people aged 15-24 years is a public health concern in Uganda. Unwanted pregnancy, unsafe induced abortions and associated high morbidity and mortality among young women may be attributed to low contraceptive use. This study aims at exploring reasons for low contraceptive use among young people.

**Methods:**

In 16 focus group discussions, the views of young people about obstacles and enabling factors to contraceptive use in Mityana and Mubende districts, Uganda were explored. The groups were homogeneously composed by married and unmarried men and women, between the ages of 15-24. The data obtained was analyzed using qualitative content analysis.

**Results:**

Young men and women described multiple obstacles to contraceptive use. The obstacles were categorized as misconceptions and fears related to contraception, gender power relations, socio-cultural expectations and contradictions, short term planning, and health service barriers. Additionally, young people recounted several enabling factors that included female strategies to overcome obstacles, changing perceptions to contraceptive use, and changing attitude towards a small family size.

**Conclusions:**

Our findings suggest changing perceptions and behavior shift towards contraceptive use and a small family size although obstacles still exist. Personalized strategies to young women and men are needed to motivate and assist young people plan their future families, adopt and sustain use of contraceptives. Reducing obstacles and reinforcing enabling factors through education, culturally sensitive behavior change strategies have the potential to enhance contraceptives use. Alternative models of contraceptive service delivery to young people are proposed.

## Background

Half of the world's population is in or entering their child bearing years. Consequently there is tremendous need for contraceptive use, especially in areas with high fertility[1]. This is particularly true in Uganda where the persistent high fertility (6.7 children per woman) is contributing to the high maternal morbidity and mortality (435/100,000 live births) as well as the rapidly growing population (3.2%) [2-4]. By comparison, a woman in two neighboring countries Kenya and Zimbabwe will have an average of 4.5 and 2.8 children in her lifetime respectively [5]. Maternal mortality is further increased by unintended pregnancies resulting in unsafely induced abortions[4]. High fertility and high maternal morbidity and mortality not only strain individuals, families, and public resources, but also hinder opportunities for economic development[6]. Use of contraceptives have the potential to avert unplanned births, decrease maternal morbidity and mortality, increase welfare and protect future generations[6,7].

In 2009, 49 percent of the Ugandan population was below 15 years and 20 percent was between the age of 15 and 24[5]. A large number of young people in Uganda are thus in or soon reaching their reproductive age and thus have a potential risk of unplanned and unwanted pregnancy [2]. By 15 years of age, eleven percent of adolescents in Uganda have initiated sex and by 18 years 64 percent of young people have had their first sexual encounter [2]. At the same time the bio-social gap has widened, with declining age at puberty and rising age of marriage[8]. Young women are thus exposed to the risk of pregnancy before marriage for a longer period and as a result there is increased need for contraceptive use. Twenty-five percent of all pregnancies are among teenagers[2]. Additionally, the birth interval is very short with 41 percent of women 15-19 having another child in less than 24 months [2]. Unplanned pregnancies constitute 46 percent of all pregnancies. Young women between 15-24 years account for nearly half of all maternal deaths due to unsafe induced abortions, which is an indication that contraception is needed [9-11].

Despite Uganda's liberal family planning policy, which states that all sexually active men and women should have access to contraceptives without need for consent from partner or parent, contraceptive use remains low, one of the lowest in the world. Awareness of contraceptives is almost universal, with 97.5 percent of people in reproductive age being able to identify at least one contraceptive method [2]. But only eight percent of married women aged 15-19 and sixteen percent of those aged 20-24 use modern contraceptive methods. Five percent of married youth aged 15-24 rely on traditional methods. Furthermore, 63 percent of sexually active unmarried women 15-19 years and 43 percent of sexually active unmarried women 20-24 years are not using any contraceptive method at all [2,12]. Condom use is low in Uganda; only two percent among married women aged 15-24 use condoms. It is worth noting that contraceptive use is two times lower in rural compared to urban areas[2]. There is a mismatch between the desire to restrict birth and the actual use of contraception despite the wide spread knowledge and efficacy. Two of five young women aged 15-24 want to space or limit childbirth but are not using contraceptives[13].

Quantitative demographic and facility-based previous studies in Uganda and other countries have identified limited contraceptive method choice, high costs, misinformation, pronatalism, constraints of women's decision-making and negative provider attitudes as barriers to fertility regulation [13-16]. However, these quantitative studies have not provided a comprehensive understanding of the various reasons for not using contraceptives as stated by the young people themselves. Data from the Uganda Demographic Health Survey though comprehensive, largely provides information on married individuals and, does not cover unmarried adolescents. There is absence of data that fully explains the knowledge practice gap for young people. This study provides an opportunity to explore what happens among both married and unmarried men and women. It also explores the gaps in knowledge and practice. Program efforts and commercial marketing strategies in Uganda have not yielded plausible rise in contraceptive use [17]. Previous studies have recommended exploration of barriers to contraceptive use among young people that would explicitly explore he factors in decision making process about use, or none-use of forms of contraceptives [18,19]. This article reports on qualitative research that explored views about obstacles and enabling factors for contraceptive use among young people aged 15-24 in two districts in Uganda. It is intended that this research will inform development of culturally sensitive policies and programs responsive to young people's needs.

## Methods

### Study settings and participants

The study was conducted in March, April and May, 2008 in Mityana and Mubende districts in the South Central part of Uganda. Mityana has a more urban and Mubende a more rural population. In 2002, the population of Mubende was 436,493, and in Mityana it was 269,763 with an annual population growth rate of 3.6 percent [20]. In 2010 the population is estimated to be 579,200 and 357,700 for Mubende and Mityana respectively. More than 60 percent of the population in the two districts is under 18 years. In these two districts, 30 percent of all pregnancies are among teenagers compared to the national average of 25 percent [2,21]. There are five main ethnic groups in these districts; Baganda, Banyoro, Ankole, Bakiga and Banyarwanda. The primary language is Luganda. The main economic activity is subsistence farming. Each district has three health sub-districts (HSD) each covering approximately 100,000 people. One HSD in each district has a hospital in addition to primary health care centers and private clinics. In each of the two districts, the HSD with a hospital was selected for the study site, Mityana South in Mityana and Buwekula in Mubende.

Married and unmarried men and women aged 15-24 living in the two study HSDs were purposively selected for the study. Purposive sampling ensured diversity in terms of age, marital status, education and socio economic background of participants in order to get various views, opinions and experiences on use of contraceptives among young people. Furthermore, participants from both rural and urban areas were selected in order to ensure as broad perspectives as possible.

### Study design and data collection

This was an exploratory, qualitative study. Focus group discussions (FGD) were used to collect data. The first author (GN), together with the local council authority in each district, identified and collaborated with village youth leaders to recruit participants. A total of 16 FGDs (8 in each HSD) with 6-12 persons in each group were conducted in the local language. The first author moderated 12 of the FGDs. The other four were moderated by a trained and experienced nurse-midwife assisted by either a male or a female social scientist with field experience. There were no differences by the moderators in guiding the discussions, an FGD guide was used. The discussions were audio taped after obtaining consent from the participants. The FGDs lasted between 60 and 90 minutes. A total of 146 young persons, 72 men and 74 women participated. Background characteristics of the participants are presented in Table 1. The FGDs were homogenously composed according to sex, age, and marital status. The discussions were held at a convenient venue identified by participants. A FGD guide developed by the research team (co-authors) was used. The research team comprised of two nurse-midwives and two obstetricians/gynecologists with public health focus. The discussions included the following topics: contraceptive practices and attitudes to contraception, contraceptive method-specific problems, perceptions of access to services, concerns about side effects, decision making process in relation to contraceptive use, and specific enabling factors and obstacles to contraceptive use. Following each discussion the moderator gave participants information about contraceptive methods and advised participant in need of methods to visit local staff at the nearest contraceptive delivery point for follow-up.

**Table 1:** Composition of the focus groups and selected individual characteristic of participants

Number of FGDs	Gender, age, and marital status of participants	Education level	Occupation
**2**	Male unmarried 15-19 (N = 19) Mityana(9), Mubende(10)	Primary, Secondary 10/9	Students (9), farmer(5), builders(1), 'boda'*(1), trader (3)

**2**	Male unmarried 20-24 (N = 17) Mityana(9), Mubende(8)	Primary Secondary 10/7	Boda (3), farmers(7), trader(1), students(5), builders (1)

**2**	Female unmarried 15-19 (N = 19) Mityana(9), Mubende(10)	Primary Secondary 9/10	Students(11), farmers (5), trader (1), None (2)

**2**	Female unmarried 20-24 (N = 15) Mityana(8), Mubende(7)	Primary Secondary 9/6	Farmers(10), traders(3), students(2)

**2**	Male married 15-19 (N = 17) Mityana(7), Mubende(10)	Primary Secondary 12/5	Farmers(12), trader(3), builders(1),Boda (1)

**2**	Male married 20-24 (N = 18) Mityana(8), Mubende(10)	Primary Secondary 12/6	Farmers(5), trader(6), Boda (5), teachers(1), carpenter (1)

**2**	Female married 15-19 (N = 20) Mityana(10), Mubende(10)	Primary Secondary 12/8	House wife(6), farmers(5), trader(7)

**2**	Female married 20-24 (N = 20) Mityana(10), Mubende(10)	Primary Secondary14/6	House wife(9), farmers(11)

* Boda boda- commercial motor cycle riders.

### Data management and analysis

The FGDs were translated and transcribed into English directly by the first author and a social scientist with experience in translation and transcription of focus group data. The first author read through all the transcripts several times while making notes on the transcripts to make sense of the texts. Each of the other three co-authors read some of the FGD transcripts. Coding of the data and the analysis was done manually. The unit of analysis was the focus group. Latent content analysis technique that involves in-depth interpretation of the underlying meanings of the text and condensing data without losing its quality was used [22]. Events, activities, perceptions and explanations were identified and coded by the first author. The codes were grouped into subcategories, categories, and then themes were identified as highlighted by Graneheim and Lundman [23]. The analysis was discussed among the research team members and discrepancies on coding and other issues that required clarity were settled by discussion. Quotes that best described the various categories and expressed what was aired frequently in several groups were chosen.

All young people provided consent either written or verbal, following the first author reading them information related to their participation. Parental or spouse permission was obtained from participants 15-17 years. The ethics and research committee of Faculty of Medicine Makerere University and Uganda National Council for Science and Technology approved the study. Administrative clearance was sought from the district authorities. Participation in the study was voluntary and confidentiality was assured.

## Results

Each FGD was unique, reflecting the variation among the two age groups (15-19 years and 20-24 years), sex and marital status. Two consistent themes emerged in all the groups, "obstacles to contraceptive use" and "enabling factors to contraceptive use". The findings are summarized and relevant quotations are used to enrich the results.

### Obstacles to contraceptive use

Various obstacles that impede contraceptive use were identified and classified into five categories that included misconceptions and fears, gender power relations, socio-cultural expectations and contradictions, short term planning, and health service barriers (See Table 2).

**Table 2:** Example of step-wise analysis for the theme: Obstacles to Contraceptives Use.

Codes	Sub-categories	Categories
Pills burn woman's eggs		
Pills pile up in body cause swellings, cancer, spoils tubes		
Oral methods are abortifacient, and cause birth defects		
Coil pierces womb		
Condom damages womb, grooves porous, lubricant infectious		
Condom break, get stuck, may cause death	Misconceptions	Misconceptions and Fears
	
Side effects are permanent		
Contraceptives cause infertility, guilty of not bearing children		
Lived or peers experiences of side effects	Fears	
Condom faulty- 'whites' insert HIV in condom		
Contraceptives make women sick and fail to work		

Men reject discussion, disapprove contraceptives use		
Men abusive, violent, abandon women using contraceptives		
Women need men's permission	Power relation	Gender power relations
Men intimidate, manipulate girls into sex without contraceptives		
Men use pregnancy to control and attach women in relationships		
	
Couple disagree on use, consensus rare		
Men decide on number of children, when to use a method or not	Decision making	
Male persuasive, girls are shy, ignorant and weak to negotiate for use	and negotiation	
Men take contraception lightly consider it a woman's problem		

Women's purpose in life is child bearing		
Women face dilemma, men demand gender distribution of children	Social norms and	Socio-cultural expectations
Society condemn early sex, pregnancy, contraceptive use by young people	expectations	and contradictions
Contraceptives for married with enough children		
Men want children from different wives		
Parents, partners, in-laws have strong negative feelings on contraceptives		
Religion oppose use of contraceptives		
	
Society links contraceptive use to promiscuity		
Women fear to be seen at health center or shop seeking contraceptives		
Young people using contraceptives are spoiled	Stigma	
Embarrassing to attend FP clinics or to buy condoms		
Men suspicious if women brings or knows how to use condom		
	
Men refuse contraception, but say children are women's responsibility		
Parents not open on sex matters, but scare youth from contraception		
Parents angered when find girls or boys with contraceptives	Contradictions	
Parents force girl/boy to marry early if pregnant		
Abortion option for girls not to have a baby		
Religion view contraception as murder, a sin		

Pre-marital sex not a problem, men/women start sex early		
Apparent trust, men develop trust in women quickly, rarely use FP		
Girls perceived to be too young to get pregnant		
Male partner's duty to use protection		
Contraception not priority, and preference of sex without condom	Emerging attitudes	Short term planning dilemma
Perceived love of a girl abandon condom use		
Pregnancy is a means to acquire a partner for marriage		
Poverty-sex with older men and commercial sex		
	
Sex done in a hurry, no time to get condom after convincing the girl		
Girls want sex, are not patient during courtship, abstinence outdated	Immediate gratification	
Young men compete for girls, use pregnancy to win them over		
Pleasure from sex overrides fear for pregnancy		

Cost burden for transport and contraceptives		
Use of condoms expensive with consistent partner		
Limited method to choose from and irregular stocks	Uncertainty for continued use	Health systems barriers
Long distance to health center, queue and waiting time, shops close early		
Providers too busy with sick people, seeking service is not convenient		
	
Provider dictate methods for young people, not friendly		
Provider advise on abstinence and not contraception		
Providers report young people seeking contraception to parent or partner	Provider paternalistic attitudes	
Fear to collide with parents at Health Center		

#### Misconceptions and fears

Misconceptions surrounding contraception and reproduction were mentioned in all FGDs. The young people believed that contraceptives interfered with fertility, and they were frightened to use something that could harm their ability to reproduce. Most of the married and unmarried women believed that pills burned the woman's eggs. Both male and female participants believed that pills accumulate in the body causing swellings, such as fibroids, cancer, destruction of the fallopian tubes, and when used they are abortifacient. Participants were also convinced that the intra uterine device could pierce the uterus.

*"Those pills are dangerous; they go through the fallopian tube and go to that area where eggs come from. So, when the pill falls in the middle of all the eggs, it burns them all.....they burn the entire woman's eggs and form a big scar. You may die without ever becoming pregnant" *(Married women's group, 15-19 years)

*"Family planning causes abnormal swelling in the uterus and cause cancer... my in-law was using these methods, and she was operated because of this. They removed fat and contraceptives ...when you take pills for a long time they pile in your body...that is why I can't use family planning" *(Unmarried woman, 15-19 years)

Condoms were believed by both male and female participants to damage the uterus, to get stuck in the reproductive tract and cause death, not to fit properly, to be porous, and to have infectious lubricant. Male participants reported tension and suspicion when using condoms because they thought 'Whites' had infected condoms with HIV. Men were said to have lost confidence in condoms supplied by the government when faulty Ngabu brand of condoms were recalled in 2007. Others believed that the oil on the condoms was infectious to women and feared that condoms had pores or grooves with actual perforations that allowed transmission of HIV.

The participants reported how fear immobilized them in their decision-making. They were afraid of the side effects, afraid of getting pregnant without contraception, afraid of the response from their church, and afraid of parent and family reactions. Fear of partners' and parents' reaction was an obstacle to contraceptive use expressed by many participants.

*"It is very hard to take pills every day when you still stay with your parents unless you keep them with friends...parents check suitcases. The person who gave birth to you, if she tells you that something is bad it is bad, parents have a lot of experience, you have to comply with them" *(Unmarried women's group 15-19 years)

The participants seemed to worry about the impact of side effects and feared that the side effects were permanent. The fear came from their own and their peers' experiences, and from misinformation given to them by parents/elders to discourage them from having intercourse. Some young unmarried women expressed more fear of the side effects than of pregnancy.

*"We want the injection method but it has many problems like palpitation, craving for foods like pregnant woman, and dizziness that can even make you sometimes fall in the garden...we work for long hours in the sun without a meal... pills can make you too weak to work, you eventually stop using ...it is safer to just get pregnant, have a baby *(Married women's group 20-24 years).

#### Gender-power relations

The groups revealed gender inequalities in terms of power, roles, decision making, and negotiation for contraceptive use. The women reported lack of power in decision making as a key obstacle to use. Both men and women reported that women's purpose in marriage is to produce children. The women recounted partner disapproval and verbal or physical abuse including violence if the man discovered that the woman used contraceptives. A man could abandon his girlfriend or wife if she insisted to continue using contraceptives.

*"If I ever find my wife using this family planning we would separate, send her away... I have heard very bad things about them." *(Married man, 20-24 years)

The women said that initiating a discussion about contraception was generally considered unacceptable, and often the partner rejected such discussions. The young married female participants reported that conflict with the husband and the in-laws was severe when family planning issues were raised. In some male groups, the participants commented that women also oppose contraceptive use and react negatively when men raise contraceptive issues, contending that women fear the risk of not having children following use of contraceptives. Women in polygamous relationships were said to compete for children.

Male participants said that women are weak and easily influenced to have unprotected sex. In contrast females reckoned that men want more children and manipulate women into sexual relations without contraceptives. The women further noted that men who do not want to use contraceptives are inconsiderate of the woman's future, citing that men make women pregnant to make them dependent. Unprotected sex for men enhanced their reputation among other young men in contrast to the situation for women. But men would deny the pregnancy and blame the woman for becoming pregnant. However, some female participants recounted that early pregnancy was perceived as a positive incentive for early marriage and was no longer perceived as a problem. Some girls were said to pierce condoms to get pregnant to compel their partners to marry them.

*"Some woman may pierce the condom with long nails with intention to get pregnant... as long as you get pregnant you no longer waste time...you just go with the man responsible for the pregnancy and start a family" *(Unmarried women's group, 15-19 years)

Some married women said they still needed permission from their husband to use contraception, and could be abandoned if it was done without his knowledge.

*"Most men do not like contraceptives, they want children, he gets a baby with you and asks you for another....if you tell him about contraceptives he leaves you....so women use it secretly" *(Married women's group, 15-19 years)

*"One friend was using 'family'-injection without approval of her husband but started bleeding severely and had to tell him to get help to go to the health unit. This caused serious problems for her since her husband wanted more children" *(Married woman, 20-24 years)

Both male and female participants expressed views about decision making, and cited power struggles, couples disagreements and rare consensus on contraceptive use. Attempts to negotiate use frequently ended in violence or separation. The final decision depended on the preferred number of children by the man. Men were also reported to make decision to use or not use condoms. Men had the view that women were given to them when they married and the women were obligated to produce children or be thrown out of the house.

*"Some men do not want their wives to use contraceptives and are very tough on that. This can even lead to separation...I heard of a scenario where the man reproached the wife who had told him that she was tired of having children. The man said he could still look after his children and wanted more" *(Unmarried man, 20-24 years)

On the other hand, some female participants mentioned that some married women with children could actually discuss with their partner and agree on use.

*"Some women talk with their partners, some men also notice the finance problems and observe how difficult it is to raise the children and agree to use of family planning" *(Married women's group 20-24 years)

#### Socio-cultural expectations and contradictions

Most male and female participants reported that the purpose of life for women is child bearing but also that traditional societal norms prohibit sexual activity and pregnancy before marriage. Furthermore, cultural norms condemn parents talking with their children about sex. Society expects young people to be virgins till marriage, but many young people reported that in reality they were sexually active. At the same time societal norms/values do not support use of contraceptives and parents were said to use scare tactics about contraceptives for example; contraceptives lead to developing of fibroids and infertility to keep their children from having sex. Parents reject contraception and yet they do not want their unmarried daughters to become pregnant. If an unmarried daughter gets pregnant, the parents might send her away from home, force her to marry the man responsible for pregnancy, or force her to undergo an abortion. The parents may also compel the young man responsible for pregnancy to leave the community, or try to send him to jail.

*"Parents and elders are against contraceptives. If they find you with a condom, they lose confidence in you...parents should be made to accept that things are changing to allow these methods...nature is nature, young people need sex, it is better to tell them about contraceptives" *(Unmarried men's group, 20-24 years)

Contradictive messages from partners, parents, clergy, teachers, cultural leaders and health workers were identified as key obstacles to use of contraceptives. Young people get mixed messages since the social norms condemn sex, contraceptives and pregnancy before marriage while partners, peers and media encourage sex. The participants said that the Churches added to their confusion with messages of opposition or silence. They all noted that the churches are very pro-natal with statements like "go out and multiply", "contraceptive use is murder" and "children are a blessing".

*"The pastor in our church emphasizes that use of family planning methods is killing, it is a big sin in front of God*" (Married man, 15-19 years)

It was noted that young people are stigmatized if they use contraceptives. Contraceptives were perceived to be for married couples who have had a number of children. Communities were said to link use of contraceptives to promiscuity and prostitution, and also to future infertility. These values placed young people in a dilemma. The young women also reported experiencing this stigma at points of contraceptive delivery. They revealed fear of being reported to husband/parent if found at health facility or at shops seeking for contraceptives. Unlike men, women were ashamed to buy or carry condoms, it was considered unacceptable. In addition, women were reluctant to seek information about contraceptives since men might become suspicious if a woman knew how to use condoms.

#### Short term planning

Peer pressure and pleasure from sex was said to override fear for pregnancy, HIV and other sexually transmitted infections. Some male participants said there was no need to use contraceptives with young women who look healthy since there was no apparent risk.

*"Girls looking beautiful and healthy, there is no method used...even no HIV test is done we check with our eyes....you look at her and determine if she is okay."*(Unmarried man, 20-24 years)

The men stated their preference for sex without a condom claiming that sex was not sweet in 'plastic wrapping'. Male participants further connoted that consistent use of condoms was difficult with stable partners they trusted and loved. Condoms were frequently abandoned after two or three encounters because the couple built trust and confidence in their relationship early.

*"You may use condoms sometimes in an extra marital affair but it reaches a time when you stop....woman view using a condom as a sign that she is not clean...over time you stop, also condoms are expensive to obtain...time taken to stop using the condom depends on the individual....till when you 'trust' her" *(Married men's group, 20-24 years)

Young men cited reasons for not using contraceptives, for example not having time to find or purchase commodities after convincing the young woman to have sex. The men explained that they usually had to make love quickly outside in the bushes or at the disco and that they were not prepared with contraceptives. Some male participants revealed that women were not patient during courtship. When the man wanted to delay sex, the woman might think that he was not serious about the relationship. Furthermore, poverty and need for money was reported to override need for protection. Male participants reported competing economic demands where they would rather spend money on new shoes or clothes than on contraceptives. It was also mentioned how difficult it was for young women to use contraceptives or negotiate for their use with older men. Similarly women involved in exchange of sex for money, or other incentives had difficulties negotiating contraceptive use. Sex for money in Ugandan context is not only by regularly known sex workers it mostly sporadic.

#### Health service barriers

Paternalistic, judgmental views by contraceptive service providers, coupled with lack of privacy and confidentiality, were said to inhibit young men and women from seeking contraceptive services and using contraceptives. Multiple examples of providers reporting to parents or husbands if young men or women came to the health unit for contraceptives were given in the focus groups.

*"If you come for condoms every day at the health unit, the health worker gets scared that you are having too much sex and can tell your parent. Youth are shy and fear to be reported to their parents" *(Unmarried men 15-19 years)

In addition, the clinics were habitually out of stock of contraceptives and had limited choices of methods, making it very difficult to use any method consistently. High costs, particularly in rural areas, were reported as an obstacle by both young men and women. Some male and female participants said that cost was a burden, making contraceptive use dependent on disposable income. Access was further restricted by cost implications in terms of transport, and distance to health facilities.

*"Some women agree on use with their partner but the contraceptives are expensive ...people are too poor here ...sometimes you can fail to get them in the near clinic, you have to spend on transport to town...it is very expensive... you may end up defaulting and getting pregnant" *(Married women's group, 20-24 years)

Limited opening hours, long waiting time, and lack of youth friendly services were other barriers to access that were mentioned. This resulted in incidents of self prescription identified among participants. Young people also felt ashamed and reluctant to ask for contraceptive services from busy health care professionals attending to sick people. Fear of stigma at the health facility propelled some women to procure contraceptives from private clinics/stores since they provided privacy and confidentiality. Furthermore, young women would sometimes buy commodities from private clinics and drug stores with unqualified staff, which sometimes was said to result in health problems and thus, in discontinued use.

### Enabling factors for contraceptive use

The key enabling factors to contraceptive use mentioned during the FGDs were classified into three categories including female strategies to overcome obstacles, changing perceptions, and changing attitude towards a small family size (See Table 3).

**Table 3:** Step-wise analysis for the theme: Enabling Factors to Contraceptives Use

Codes	Sub-categories	Categories
Secret use of contraceptives, fear of male partner opposition and stigma		
Sneak to Health units for contraceptives		
Contraceptives bought private units to cope with stigma		
Know some contraceptive methods and cost	New Coping strategies	Female strategies to overcome obstacles
Some women convinced of contraceptive benefits		
Some women carry condom in case of emergency		
Use contraception to limit pregnancy in unstable relation		
Self preservation for women with marriage intentions		

Fear pregnancy more than HIV/AIDS		
Fear burden of carrying pregnancy, and costs involved		
Fear of abortion complications		
Men fear jail, forced migration if make a young girls pregnant	Risk perception	Changing perceptions
Pills back up when condom not used		
	
STI prevention with secondary contraceptive value		
Condom used when suspicious of girl		
No permission is required for men to use condoms	Selective use	
Contraceptive use reduce spending		
Economic status influence number of children		

Belief in small family to sustain good life		
Value of children education, health care expenses		
Fear early family		
Struggle to survive, children are a burden	Small family size	Changing attitude towards a small
Some men fear responsibility, welfare of children		family size
Many children affects employed men and women		
Young people debates encourage few children		
Choice of long acting method in stable relationship		

#### Female strategies to overcome obstacles

Married and unmarried young women were aware of different contraceptive methods, their cost, and some were also convinced of the benefits of using contraceptives. Some women decided to use or not to use contraceptives independently from their partners. Some unmarried women also reported that they carried condoms in case of emergency in order to avoid pregnancy in unstable relations. Young women recounted using contraceptives secretly as a strategy to protect their interests, and counteract male partner disapproval. Secret use of contraceptives was said to prevent unwanted pregnancy. At the same time conflicts with partners and families were avoided. Secret use was an enabling factor specific for young women.

*"For me I have to take the pills secretly as I study the man I love, because he might leave you if you become pregnant and then you begin thinking of abortion... that is why most youth hide first time using contraceptives" *(Unmarried women's group 15-19 years)

Some female participants, regardless of marital status, reported going secretly to health facilities for contraceptives. As a result the injection method was said to be the most common method used both by married and unmarried young women.

*"Some married women use contraceptives in secret, most men don't like contraceptives and refuse women from using. Pregnancy is so stressful for women, your husband may not be supportive financially but only interested in alcohol...you need to work and save money from farming for your children, so you sneak in the health unit for injection when coming from the garden" *(Married women's group, 20-24 years)

#### Changing perceptions

Male and female participants reported that they feared pregnancy more than HIV/AIDS, contending that HIV/AIDS is like having a common fever. Participants in the focus groups claimed that previously people were more scared of HIV than pregnancy. Fear of pregnancy and not HIV was reported to slowly aid adoption of contraceptive methods. HIV/AIDS prevention campaigns emphasizing condom use were reported to improve decision-making towards use of condoms for prevention of pregnancy as well.

*"Young women fear pregnancy not AIDS, they consider the burden to carrying pregnancy for nine month, costs involved during pregnancy and when baby is born...they fear pregnancy but HIV who knows when you get it... AIDS takes long to show compared to pregnancy so you would rather have that than pregnancy that shows soon" *(Unmarried women's group 15-19 years)

Female participants reported that self preservation from pregnancy before marriage led to contraceptive use. Furthermore, worries of carrying a pregnancy, looking after the baby, the costs involved, and fear of negative consequences of abortion were enablers to contraceptives use.

*"Pregnancy is tough, one considers cost involved in carrying pregnancy, food and others...it is so worrying to young women. Most of them fear the burden of looking after the child. Taking care of yourself and pregnancy is tough" *(Unmarried women's group 20-24 years)

Male counterparts reported fear of early forced marriage, being forced to leave school or being put in jail if found to have made a young girl pregnant as motivators for contraceptive use. Selective contraceptive use was also reported with "on and off" partners and when the man suspected that the woman had a sexually transmitted infection.

#### Changing attitude towards a small family size

Both male and female participants recognized that having many children is difficult for working men and women. This was a common view among those living in trading centers/towns and these young people were motivated to use contraceptives. Men did not want to have the responsibility of many children in terms of education and health care expenses. Young men and women stated that they often debated among peers about having fewer children.

*"There are some seminars around encouraging young people to have few children...so that kind of thinking is the one that has been common in young people's debates" *(Unmarried men's, 20-24 years)

Women also reported that there were a few men who did not want many children and demanded that women use contraception.

*"But there are some men interested in contraceptives, they ask a woman to use such that they don't have frequent birth like a rabbit... he may not manage many children, so he demands that the woman uses or else she assumes responsibility of pregnancy on her own" *(Married women's group 20-24 years)

Female participants recounted desire for a small family, and awareness of contraceptive methods, as incentives to contraceptive use. They further reported that free contraceptives in the public facilities helped use. The women who already had children also stated that antenatal clinics were an important source of information to encourage contraceptive use.

## Discussion

The results of this qualitative study elucidated a deeper understanding of why young people use or not use contraceptives. The young people in the FGDs revealed individual, socio-cultural, economic, and institutional obstacles to contraceptive use. Fifty years after introduction of family planning in Uganda, utilization is still low and there are many challenges of translating policy and knowledge into practice

The participants' perceptions indicate knowledge gaps and limited access to information as well as comprehensive family planning services. The study findings show that young people have strongly embedded misconceptions and profound fears which serve as obstacles to initiation and continuation of contraceptive use. Many perceived contraceptives to be harmful and detrimental to health, and because of this they were reluctant to use them. The reported fear of infertility is a sign of the inherent need to have children and the importance that society places on child bearing. Young people seemed to lack knowledge about how contraceptives work and had made up their own ideas about the mechanism involved. Additionally, young people placed considerable weight on the side effects of both hormonal and non hormonal contraceptive methods. Although few side effects are known to be life threatening [24], irregular heavy bleeding, loss of libido and weight gain are significant to young people, especially for women using contraceptives secretly. For many women in their prime reproductive years such side effects contribute to poor adherence and contraceptive failure. Opposition from male partners was noted to partly stem from beliefs about such side effects. A study on sterility rumors in Africa asserted that in absence of the truth, rumors acquire credibility[25]. Research studies have linked limited knowledge about contraceptive methods and concerns about health and side effects to high unmet contraceptive needs in low-income countries [26]. Young people reported how fear immobilized them in decision making. They were afraid of several issues; for example; side effects, getting pregnant without contraception, church response, partners, parents and family reactions. Another study in Uganda identified parents as an obstacle to use of emergency contraceptives [27]. It is important to provide factual, correct information to demystify the misconceptions around contraception. Adequate clinical support is required to manage side effects, without this social marketing will continue to fail for hormonal contraceptives.

Previous interventions in Uganda have been based on the belief that lack of information is the basis of low contraceptive use [17,28]. But lack of awareness is not the only obstacle to practice. The current social norms create significant conflicts, and dilemmas for young men and women, as sexual practices and life styles are changing. Our findings underline the contraceptive controversies and contradictions that exist based on powerful and persistent religious, cultural and social values. Traditions and cultural practices that encourage high fertility are still strong in Uganda, which has been reported in reproductive health symposium in Uganda [11]. Our study findings revealed that young people experienced resistance and opposition to contraceptive use by adults including religious, political, and cultural leaders. The leaders are pro-natal and spread negative messages that scare people from using contraceptives and contribute to spread of rumors, misunderstandings and criticisms in the communities. Findings from this study revealed that young women are still bound by cultural norms that equate marriage and motherhood with female status and value, creating pressure on them to prove their fertility, which was also found in a study on adolescent motherhood in Uganda [29]. Our study findings illustrates that contraceptive programs should be sensitive to culture and traditional perspectives to birth control. Success of programs will depend on responsiveness to young people's needs, use of appropriate cultural designs that promote social change while respecting the cultural values. Efforts need to focus on changing individual and societal motivations. Engaging respected community gatekeepers including teachers, parents, clergy, health care providers, local 'stars' and media into dialogue might be a plausible strategy.

Contradictive messages from male partners, parents, clergy, teachers, peers, media and health workers alongside social norms that condemn sex, contraceptives and pregnancy before marriage, imply that young people get mixed messages. Furthermore, peer pressure and pleasure from sex was noted to override fear of both pregnancy and HIV. It was also shown that young men and women feared pregnancy more than HIV infection since the later take long to develop. Similar findings were reported in a study on emergency contraceptives[27]. This shows short term thinking and is an attitude that might fuel the HIV epidemic. Our results indicated that trust was built early in relationships and that sex without condoms was preferred. Related findings were reported in a United Kingdom study on risk, power and trust which reported that young women mobilized trust in male partners as a coping strategy[30]. Condom use was mostly related to HIV prevention, which indicates that women are at risk of pregnancy as soon as the couple has overcome the fear of HIV. Young people do not have a regular sex life and their sudden unplanned sexual encounters make contraceptive use difficult. Some participants noted that young women and men were using pregnancy to attract their partners into marriage, a finding that further demonstrate young people's short term planning perspective. It is worth noting that the age of consent for heterosexual sex in Uganda is 18 years but sex is initiated much earlier. Condoms have a comparative advantage to other contraceptive methods; it can prevent both unwanted pregnancy, and STI including HIV. Promotion of condom for dual use would improve contraceptive usage rates. Use of social community networks to improve information, service and counseling might overcome socio-cultural or peer norms and foster skills in behavior change strategies.

Our results underscore how conventional gender inequalities in terms of decision making and negotiation for contraceptive use are major obstacles to contraceptive use. The participants described social cultural norms on gender roles that emphasize the man as the decision-maker in the family, including decisions on contraceptive use. Men want and demand many children, and their fertility preference promotes unilateral decision making with a negative influence on female contraceptive use. Our study highlighted limited couple discussions, frequent disagreements and partner violence when the woman wanted to initiate or continue using contraceptives. Many young women seem to see fertility as a matter outside their control because of gendered cultural structures. Most women have limited access to resources and are financially dependent on their partners[31], which enhances women's sub-ordinate position. Furthermore, women in polygamous relationships were said to compete for children as a means of security, which is another negative impact. While it will take time to change the social norms, decision to use contraceptives should be taken within a family.

Would all young men and women use contraceptives if made available to them? The answer is clearly no. There appeared to be shifting of blame between men and women. Both male and female opposition to contraception was noted although male partner opposition was more prominent due to men's power in decision making. The opposition reflected fertility preference, fear of side effects, misconceptions, and doubts in the safety of the contraceptive methods. Research have reported that men's preference influence contraceptive use more than women's preference [28]. Our study findings reveal knowledge gaps and misinformation among men. Men are equal partners and male participation in contraception programs is essential.

Unmarried women using contraceptives are stigmatized and perceived to be either prostitutes or promiscuous. In contrast, sex experience by men was said to enhances their reputation among other men, which has also been noted elsewhere [32]. Studies have demonstrated difficulties to satisfy contraceptive needs in communities where contraceptives are believed to be only for married people [33]. Increasing contraceptive use requires personalized, individualized strategies to men and women in accordance to cultural norms.

The cost for transport and contraceptive commodities, made it prohibitive, especially for those without a disposable income. Such structural impediments negatively affect contraceptive use rates among young people. Research studies have also noted how costs affect use [29,32]. Integration of contraceptive services in social congregations, schools and other communal points is a plausible alternative to reduce costs. Furthermore, young men and women identified paternalistic, restrictive and judgmental attitudes among health care providers, coupled with unfriendly services, as obstacles to receiving contraceptives. Young people who had overcome all the other obstacles were stopped at the service level. Negative response from health workers also serve as obstacles to initiation as well as to continuation of modern contraceptive methods among young people. It was further noted that providers restrict access to contraceptives to the unmarried and those with no children. Strategies to strengthen health care provider's knowledge, skills and attitudes in youth friendly services are vital. The provider's opinion influence services young people receive and their subsequent contraceptive behavior.

Enabling factors are defined as factors that make it possible or easier for the individual or population to change their behavior [34]. This study identified enabling factors to contraceptive use including female strategies to overcome obstacles to contraceptive use, changing perceptions and changing attitudes towards family size. Secret use of contraceptives by some women was a strategy identified in our study to counteract traditional and social values. A similar finding was reported in a study in Ghana [31]. The secret use demonstrates the capacity of young women to take action in contexts where social norms prevent women from using contraceptives. This shows that some women make deliberate decisions and efforts to use contraceptives. They maneuver and find ways to use contraceptives despite the gender power inequalities and oppositions from partner and parents. Injectable contraception is the easiest method to use secretly. Secrete use however, is likely to put the women at greater risk of violence and desertion. Unfortunately concealing contraceptive use generally contributes to short duration of use and high discontinuation rates [35]. Where to hide the contraceptives is usually a challenge in homes with limited space and little respect for individual privacy and might also lead to non compliance with contraceptive regimen. There is need for more dialogue and open communication to delineate the secrecy and to de-stigmatize the use of contraception for sustainable use.

Our results further illustrate a difference in motive and perspectives of young men and women to use contraceptives. Some young men who were changing attitudes towards use of contraceptives were motivated by fear of imprisonment and forced marriage in case made a young girl pregnant. On the other hand for some young women were motivated to use contraceptives to avoid the burden of pregnancy and preserve themselves for long term relations. These individual and gender differences are important factors when designing programs and interventions approaching young people.

Changing attitudes towards a small family size were noted in our study. Some young people acknowledged benefits of a small family size and the importance of education and were motivated to use contraceptives. The desire for limiting the number of children and birth spacing are changing attitudes and a positive sign showing that young people are not thinking like their parents. The participants revealed urgent need to use contraceptives amidst economic and social pressures. A research report from several developing countries noted a similar trend on adolescent's contraceptive use and discontinuation [35]. Urban residences, improving education, occupation are attributed to improved position and control of women in turn impacting on women's ability to negotiate for contraception. Antenatal clinics were reported to be an important source of information to women with children, a contrasting finding to the stigma from the community about contraceptive use discussed earlier.

The results from this study are not generalizable to all young people in Uganda but the richness of the data provides explanations and reasons for use or not use of contraceptives as stated by young people themselves. Future studies should examine providers' views as well as the quality of contraceptive services in order for the health system to be able to provide good contraceptives service to young people in need. We further suggest more research on how to approach and discuss with men about the risks of unwanted pregnancy and excessive fertility.

The implications of our findings are that alternative models of contraceptive delivery are needed for young people. Results reflect an attitude change but not indicative of broad conviction among young men and women to adopt and sustain the use of modern contraceptives. Our study illustrates that contraceptive programs should be sensitive to culture and traditional perspectives to birth control. It is vital to exploit secrete use, fear of pregnancy, and the reported fear of pregnancy but not HIV to strengthen dual method contraceptive use to ensure STI/HIV prevention with secondary contraceptive value.

## Conclusions

There are changing perceptions and a behavior shift in favor of contraceptive use and small family size among young people but the multiple obstacles are still remarkable. The intensity of misconceptions and fears is extraordinary. There is gender power inequity and men make most fertility decisions. Furthermore, strong social cultural norms, short term planning and barriers to health care access including negative response of service providers, influence contraceptive use negatively. There was urgent need and desire to use contraceptives despite the differences in motive and perspectives for young men and women. It is critical to integrate contraception programs with other strategies that promote gender equitable norms and address gender equity.

## Abbreviations

AIDS: Acquired Immune Deficiency Syndrome; FGD: Focus Group Discussion; HIV: Human Immunodeficiency Virus; HSD: Health Sub District

## Competing interests

The authors declare that they have no competing interests.

## Authors' contributions

GN conceived the study, conducted the field work, participated in transcription, analysis and writing of the manuscript. EF and FM contributed to the development of qualitative research components, analysis and contributed to writing of the manuscript. JB contributed to writing of the manuscript. All authors read and approved the final manuscript.

## Pre-publication history

The pre-publication history for this paper can be accessed here:

http://www.biomedcentral.com/1471-2458/10/530/prepub
